# The impact of the Tigray War on the incidence of low birth weight in Tigray, Ethiopia: A retrospective cohort study

**DOI:** 10.1371/journal.pgph.0006298

**Published:** 2026-08-10

**Authors:** Aregawi Gebreyesus, Mamit Gebreslassie Gbrekidan, Kahsu Tsegay, Tesfay Teklu Berhe, Tewelde Gebrehawerya, Gbreagziabher Gebrekirstos

**Affiliations:** 1 Department of Epidemiology, College of Health Science, Mekelle University, Mekelle, Tigray, Ethiopia; 2 Department of Reproductive Health, College of Health Science, Mekelle University, Mekelle, Tigray, Ethiopia; 3 Department of Emergency and Critical Care Nursing, College of Health Sciences, Mekelle University, Mekelle, Tigray, Ethiopia; 4 Department of Midwifery, College of Health Sciences, Mekelle University, Mekelle, Tigray, Ethiopia; 5 Department of Neurosurgery, College of Health Sciences, Mekelle University, Mekelle, Tigray, Ethiopia; Aga Khan University, PAKISTAN

## Abstract

War and armed conflict negatively impact neonatal health. This study investigates the effect of the Tigray War on the incidence of low birth weight (LBW) among live births in Tigray, Ethiopia. A retrospective cohort study was conducted among 1,763 mothers from December 2018 to November 2022 at two public hospitals in Mekelle. Data were extracted from maternal records using systematic sampling, and multivariable logistic regression was used to identify factors associated with LBW, with significance defined as p < 0.05 and 95% confidence intervals (CI). The overall incidence of LBW was 12.9% (95% CI: 11.3–14.5). The incidence nearly doubled from 8.6% pre-war to 17.2% during the war (p < 0.001). Residence outside Mekelle, war exposure, mode of delivery, and preeclampsia were significantly associated with LBW. Mothers delivering during the war were over twice as likely to have LBW infants (Adjusted Odds Ratio [AOR] = 2.22; 95% CI: 1.64–3.00) compared to the pre-war cohort. LBW incidence increased significantly during the Tigray War. These findings demonstrate a strong association between war exposure and adverse birth outcomes, highlighting the urgent need for targeted maternal and child health interventions in war/conflict-affected regions.

## Introduction

The Tigray War, which erupted in November 2020, has had profound and far-reaching consequences on the Tigray region [1]. This war has not only resulted in significant loss of life and displacement but has also severely impacted the healthcare infrastructure and access to essential services [2,3]. Among the myriad of health issues exacerbated by the war, the incidence of low birth weight (LBW) has emerged as a critical concern. Low birth weight, defined as a birth weight of less than 2,500 grams [4], is a significant public health issue associated with increased neonatal morbidity and mortality, long-term developmental challenges, and a higher risk of chronic diseases later in life [5–7].

Prior to the conflict, the Tigray region was characterized by a well-performing health system, with a Total Fertility Rate (TFR) of 3.7 and an institutional delivery rate of approximately 81% [8,9]. However, the onset of the war in November 2020 triggered a near-total collapse of these public health gains. Reports indicate that approximately 75% to 80% of health facilities in the region were looted, damaged, or rendered non-functional [2]. Consequently, the disruption of healthcare services during the Tigray War has led to a decline in maternal and child health services, including prenatal care, nutrition support, and access to skilled birth attendants [9,10,11]. Pregnant women in conflict-affected areas often face increased stress, malnutrition, and limited access to healthcare, all of which are known risk factors for LBW. Furthermore, the war has resulted in widespread food insecurity, which disproportionately affects vulnerable populations, including pregnant women and infants [12–14]. The combination of these factors raises urgent questions about the impact of the Tigray War on birth outcomes, particularly the incidence of low birth weight.

Different literature consistently demonstrates that prenatal exposure to armed conflict negatively impacts birth outcomes. Studies across multiple countries and conflicts show that intrauterine exposure to violence reduces birth weight and increases the likelihood of low birth weight [15–18]. The effects are particularly pronounced during early pregnancy, with first-trimester exposure having the strongest impact [16,19]. The magnitude of the effect varies, with estimates ranging from 20g to 2.8% reduction in birth weight [15,18]. Infants born to mothers from disadvantaged backgrounds are especially vulnerable to these adverse effects [15]. The mechanisms behind these outcomes are thought to involve prenatal psychological or economic stress triggered by violent conflict [18].

Understanding the relationship between the Tigray War and the incidence of low birth weight is crucial for informing public health responses and policy decisions in conflict-affected regions. As the international community continues to address the humanitarian crisis in Tigray, it is imperative to prioritize maternal and child health to mitigate the long-term consequences of the war on future generations. This research not only contributes to the existing body of knowledge on the health impacts of conflict but also underscores the importance of integrating health services into humanitarian responses to safeguard the well-being of vulnerable populations.

This study investigates the effect of the Tigray War on the incidence of low birth weight (LBW) among live births. By analyzing data from public hospitals in Mekelle, we sought to determine the degree to which conflict-related disruptions influenced birth outcomes and identify key predictors of LBW. This research provides critical evidence regarding the public health toll of war on maternal and neonatal health, highlighting the necessity of prioritized interventions for conflict-affected populations.

## Method and materials

### Ethics statement

Ethical clearance was obtained from the Institutional Review Board (IRB) of the College of Health Sciences, Mekelle University (Ref: MU-IRB 1957/2022). Formal administrative permission was also secured through a support letter from the Tigray Regional Health Bureau and the respective hospital administrations. Because this study was a retrospective review of secondary medical records (maternal delivery charts), the requirement for informed oral or written consent was officially waived by the IRB. To protect participant privacy, all data were fully anonymized and de-identified before the researchers accessed them. No personal identifiers, such as names or specific identification numbers, were extracted or stored, and all data were handled in strict accordance with the principles of the Declaration of Helsinki.

### Study setting and period

This study was carried out in two public hospitals in Mekelle city, Tigray. Mekelle is the capital city of Tigray. It has more than 510,000 population administered under seven sub-cities administration. Mekelle had one primary, two general, and one comprehensive referral hospital. This study applied a retrospective cohort study from the maternal card records review to extract data in public hospitals of Mekelle City from January 18 up to 07 April 2023.

### Population and sampling technique

The study population consisted of mothers who gave birth at Ayder Comprehensive Referral Hospital and Mekelle General Hospital during two distinct periods: December 2018 to October 2020 (Pre-war, Group I) and November 2020 to November 2022 (During-war, Group II). The estimated source population recorded in the delivery logs for these periods was 31,734.

To select the sample, we employed a systematic random sampling technique. The sampling frame was derived from the delivery registration books of each hospital. The sampling interval (k) was calculated for each facility: k = 16 for Ayder and k = 20 for Mekelle General Hospital. For each study period and facility, the first record was selected using a lottery-based random start, followed by every 16th and 20th record, respectively.

The initial sample size required was 1,526; however, to maintain statistical power despite the challenges of retrospective data in a conflict zone, we included a 15% allowance for lost cards or records with missing data (n = 237), resulting in a final analyzed sample of 1,763. Records were excluded if they were missing more than two key variables or if the newborn was diagnosed with a congenital anomaly.

### Sample size determination

The sample size was determined for both objectives using Epi-info software. Using previous studies done in different countries, we determined the sample size for the first objective, we used a single proportion formula and for the second objective, we used a double proportion formula. Finally, we have taken the sample size determined for the second objective using Al-Shahethi et al, 2023 [20] with a factor of “gravidity” (proportion of the exposed: 7.2% and proportion of unexposed: 3.8). We used the assumptions of: power: 80%, margin of error: 5%, ratio of exposure and non-exposure proportion: 1:1. Finally the sample size became 1,526 and with the 15% allowance for missed/incomplete cards (229) and we have taken the final sample size of 1763.

### Variable and measurement

The outcome variable of this study was low birth weight. The independent variables were the socio-demographic characteristics of the mother (Age, residence), gestational hypertension (eclampsia, pre-eclampsia), Sex of the newborn, pregnancy complications such as antepartum hemorrhage (APH), and exposure status of the war period.

Low birth weight is defined if the fetus is less than 2,500 grams at birth.

### Data extraction form and data collection

The data extraction form is adapted from different literature carried out across the globe. A pre-tested standard extraction form was used to collect information from cards of mothers who gave birth from December 2018- November 2022. Data was collected using the ODK online system from January 18 up to 07 April 2023 at both hospitals by trained data collectors and supervisors.

### Data quality control

All data collectors were trained on the extraction tool and objective related to the study (how each characteristic is going to be measured, how to complete the tool, and how to resolve any potential problems faced). During the data extraction process, the filled checklist was checked for completeness, consistency, and accuracy by cross-checking sample forms with patient charts. Then, the online ODK tool was used to minimize errors that happen during data entry like coding errors, unrealistic values, and inconsistent date format.

### Data analysis

Data were analyzed using SPSS version 27. The normality of continuous variables was assessed using the Shapiro-Wilk test; variables with non-normal distributions were summarized as medians with interquartile ranges (IQR), while categorical data were presented as frequencies and percentages. Incidence rates were calculated for both pre-war and during-war cohorts. Binary logistic regression was used to identify factors associated with low birth weight. Variables with a p-value <0.25 in the bivariate analysis were included in the multivariable model to ensure potential confounders were captured. In the final multivariable model, statistical significance was defined as p < 0.05. The Variance Inflation Factor (VIF) was used to assess multicollinearity, with all values remaining <5. Model fitness was confirmed using the Hosmer-Lemeshow goodness-of-fit test (p = 0.57).

## Results

### Sociodemographic characteristics

A total of 1763 medical charts of mothers were reviewed. Of those 884 medical charts were mothers delivered before the war and 879 medical charts were mothers delivered during the war. The median age (interquartile range) was 27 (23 – 30) years for mothers delivered before the war and 27 (24 – 30) years for mothers delivered during the war. The median and interquartile range of birth weight was 3100-gram (2800–3500-gram) for babies delivered before the war and 3100-gram (2600–3500-gram) for babies delivered during the war. 89.4% of the study participants reside in Mekelle. [Table 1]

**Table 1:** Sociodemographic characteristics of study participants in Mekelle, Tigray: December 2018- November 2022.

Variables	Response	Before the war n (%) n = 884	During the war n (%) n = 879	Total n (%) n = 1763
Age (yrs)	15-19	30 (3.4)	30 (3.4)	60(3.4)
	20-34	754 (85.3)	754 (85.8)	1508(85.5)
	35-49	100 (11.3)	95 (10.8)	195(11.0)
Residence	Mekelle	790 (89.4)	786 (89.4)	1576(89.4)
	Out of Mekelle	94 (10.1)	93 (10.6)	187(10.6)

### Incidence of newborn low birthweight

The overall incidence of newborns low birthweight was 12.9% (95% CI = 11.3-14.5). However, the incidence of newborns with low birthweight before the war was 76(8.6%), and during the war 151(17.2%). There was a significant difference for newborns with low birthweight before the war and during the war with a p-value <0.001. The (Mean ± SD) of the newborn’s weight was similar, 3074.9 + 598 grams with a range of 800–5000 grams before the war period, and 3030.9 + 657.3 grams with a range of 500–5500 grams during the war. [Table 2]

**Table 2:** Incidence of newborn low birthweight among study participants in Mekelle, Tigray, Ethiopia, December 2018- November 2022.

Variable	Response	Before the war	During the war	Total	p-value
		n (%)	n (%)	n (%)	
Having a low birthweight of less than 2500 g	Yes	76 (8.6)	151 (17.2)	227(12.9)	<0.001
	No	808 (91.4)	728 (82.8)	1536 (87.1)	

**Fig 1:**
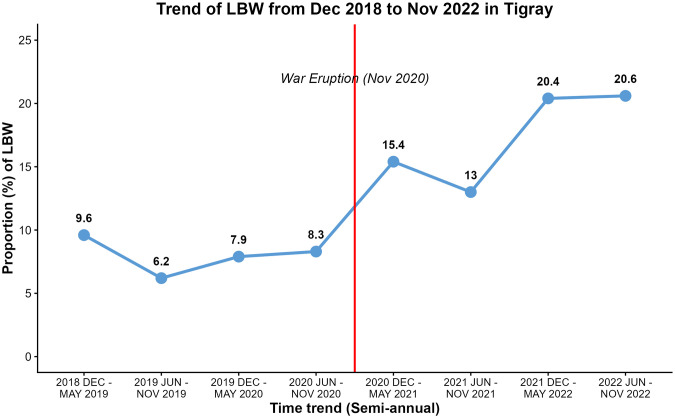
Semi-annual trends in the proportion (%) of low birth weight among live births in Mekelle public hospitals, Tigray (2018–2022). The line graph illustrates the percentage of infants born with low birth weight (LBW) across eight semi-annual periods. The vertical red line indicates the onset of the Tigray War in November 2020, distinguishing the pre-war period from the active conflict period. LBW is defined as a birth weight of less than 2,500 grams.

As illustrated in Fig 1, the proportion of newborns with low birth weight was relatively stable prior to the conflict, ranging from 6.2% to 9.6%. Following the eruption of the war in November 2020, there was a sharp and sustained increase in LBW, peaking at 20.6% by late 2022. [Fig 1]

Among newborns delivered before the war, 83.8% were categorized as having normal weight, while the proportion decreased to 76.3% during the war. The frequency of moderately low birth weight rose significantly during the war, from 10.2% to 16.7%, suggesting an increase in adverse birth outcomes during this period. Similarly, macrosomia (newborns with higher-than-normal birth weight) slightly increased from 4.4% before the war to 5.3% during the conflict. The frequencies of very low birth weight (1.5% before the war and 1.4% during the war) and extremely low birth weight (0.1% before the war and 0.2% during the war) showed minimal variation. These findings indicate a notable shift in the distribution of newborn weight categories during the war period, reflecting the broader health and nutrition challenges faced by pregnant women in conflict-affected settings [Table 3].

**Table 3:** Newborn weight category in Tigray Public hospitals from december 2018- november 2022.

Newborn Weight Category	Before the war		During the war	
	Frequency	Percent	Frequency	Percent
Normal Weight	741	83.8	671	76.3
Macrosomia	39	4.4	47	5.3
Moderately low birth weight	90	10.2	147	16.7
Very low birth weight	13	1.5	12	1.4
Extremely low birth weight	1	.1	2	.2
Total	884	100.0	879	100.0

### Association of war and low birthweight

Bivariate logistic regression analysis was performed between independent variables and newborn low birthweight. The finding revealed that exposure status during the war period, mode of delivery, antepartum hemorrhage, and preeclampsia were statistically significant with newborn low birth weight in the bivariate model. In the multivariable logistic regression, residence, exposure status of the war period, mode of delivery, and preeclampsia were found to be significantly associated with low birth weight. After controlling other variables such as residence, mode of delivery, and preeclampsia, mothers who gave birth during the war period were two times more likely to deliver low birthweight babies as compared to mothers who gave birth before the war AOR = 2.22(95% CI, 1.64,3.0). [Table 4]

**Table 4:** Bivariate and multivariable logistic regression analysis on association of war and newborn low birthweight in public hospitals of Tigray: December 2018- November 2022.

Variables	Response	Having low birthweight		COR (95%CI)	p-value	AOR (95%CI)	p-value
		Yes (n)	No (n)				
Residence	Mekelle	187	1389	**1**		**1**	
	Out of Mekelle	40	147	2.021(1.38,2.96)	<0.001	1.91(1.29,2.84)	**0.001**
Exposure status of the war period	Before the war	76	808	**1**		**1**	
	During the war	151	728	2.205(1.65,2.96)	<0.001	2.22(1.64,3.0)	**<0.001**
Mode of delivery	SVD	140	1119	**1**		**1**	
	C/S	86	396	1.74(1.3,2.3)	<0.001	1.66(1.23,2.25)	**<0.001**
	Instrumental delivery	1	21	0.38(0.051,2.85)	0.347	0.35(0.05,2.67)	0.31
Antepartum hemorrhage	Yes	19	52	2.61(1.51,4.5)	<0.001	1.3(0.7,2.41)	0.41
	No	208	1484	**1**		**1**	
Preeclampsia	Yes	26	33	5.89(3.45,10.06)	<0.001	5.1(2.86,9.15)	**<0.001**
	No	201	1503	**1**		**1**	

N.B. COR = Crude Odds Ratio, AOR = Adjusted Odds Ratio, **1** = Indicates reference group.

## Discussion

This study aimed to assess the incidence of low birth weight and the association between the war and newborn low birth weight. Based on this finding, the incidence of low birth weight before the war was 76(8.6%), and during the war 151(17.2%), and the overall incidence was 12.9%. The exposure of war, residence, mode of delivery, and preeclampsia became significantly associated with low birth weight.

In this study, the low birth weight during the war is higher compared to before the war. This incidence is much higher than the findings that were studied in Tigray before the war (14.3%) [19], and Tigray (14.6%) [21]. Southeast Asia region (12.2%) [22]. However, this result was lower than the previous study conducted in India [23] 18.2% and South Sudan 28.9% [24]. During the 1992–1995 war in Bosnia and Herzegovina, the incidence of low birth weight infants increased significantly compared to pre-war and post-war periods [25].

The finding of this study showed that exposure status during the war period was significantly associated with low birth weight. Mothers who gave birth during the war period were two times more likely to deliver low birthweight babies as compared to mothers who gave birth before the war AOR = 2.22(95% CI, 1.64,3.0). This might be due to conflict is known to cause poverty and food insecurity. This in turn may lead to nutritional deficiencies that are unfavorable to the fetus [26–28]. In addition, in war, the mother’s stress has been linked to excess production of the placental-derived corticotrophin-releasing hormone that accelerates the maturation of the fetus’ organs and increases the risk of low birth weight, especially if exposure happens during the first trimester [25,29]. Such stress responses may come from losing partners, other family, and the community. Conflict often reduces healthcare access, including the use of prenatal care and institutional child delivery [30].

Multiple studies indicate that armed conflict and war have significant negative impacts on newborn health, particularly birth weight. Studies across multiple countries show that prenatal exposure to conflict, especially in early pregnancy, reduces birth weight and increases the likelihood of low birth weight [15,16,31–33]. In the Democratic Republic of Congo, maternal war stress was strongly associated with lower newborn birth weight and epigenetic changes [34]. Similar effects were observed in Ukraine during the recent conflict with Russia, where the frequency of low birth-weight newborns increased from 5.99% in 2021 to 6.09% in 2023 [35]. The negative impacts of war on newborn health are more pronounced in regions experiencing active hostilities and temporary occupation [35]. Additionally, infants born to mothers from disadvantaged backgrounds, such as those with low education or income, are particularly vulnerable to these adverse effects [15,33].

According to this study, women having gestational hypertension/preeclampsia were five times more likely to deliver low birth weight compared to women with no preeclampsia, consistent with a study conducted in Peninsular Malaysia [19] and reported meta-analysis [36] Ethiopia [37]. This may be associated with oxygen and nutrients supplied through the placenta to the fetus becoming compromised as a result of vasoconstriction of blood vessel walls during a hypertensive state, and uteroplacental insufficiency causes intrauterine growth retardation, resulting in low birthweight [38,39].

Our finding that mothers residing outside of Mekelle were nearly twice as likely to deliver LBW infants (AOR = 1.91) suggests that selection bias may actually lead to an underestimation of the true war impact. Women in rural areas, who faced the most severe food insecurity and healthcare total collapse, were the least likely to reach these hospitals. Therefore, the community-wide incidence of LBW in Tigray is likely even higher than the 17.2% recorded in this study. This is consistent with the study done in Malaysia [40], and Tigray [21]. This could be due to the accessibility of medical services, health information, and nutritional awareness which are more prominent as the woman resides in urban areas than rural areas.

### Strengths and Limitations of the study

The strength of this study was the sample was representative because the sample size was adequate. The study used a comparative group which is prewar and during war, so the information obtained during wartime may be helpful for wartime intervention.

This study has several limitations. First, as a hospital-based retrospective study, the findings may not be fully generalizable to the entire population of Tigray. During the war, a ‘survival bias’ or ‘selection bias’ may have occurred; mothers with the least resources or those in areas under active combat may have been unable to reach Mekelle for delivery. If these excluded women experienced higher rates of malnutrition and stress, our study might under-represent the true severity of the LBW crisis. Conversely, if the hospitals primarily captured high-risk referrals, the incidence might be higher than the community average. However, the consistency of our findings with the timing of the blockade (Fig 1) and the significant association with rural residence (Table 4) provide strong evidence that the war was a primary driver of the observed outcomes.

## Conclusion

This study reveals a significant increase in the incidence of low birth weight during the Tigray War, rising from 8.6% before the conflict to 17.2% during the war. The findings indicate a strong significant association between exposure to war and low birth weight, highlighting that mothers who gave birth during the conflict were more than twice as likely to have low birth weight infants compared to those who gave birth prior to the war. Furthermore, the significantly higher risk observed among mothers from outside the regional capital suggests that the impact of the war on neonatal health was most severe in rural areas where access to humanitarian aid was most restricted. Public health responses in war and conflict zones should prioritize mobile nutritional support and blood pressure monitoring for pregnant women to mitigate these risks.

## Supporting information

S1 File STROBE checklist for cohort studies. Reproduced from the STROBE Statement under a Creative Commons Attribution 4.0 International License (CC BY 4.0).
(PDF)

## Abbreviations

LBW: Low Birth Weight
APH: Antepartum Hemorrhage
SVD: Spontaneous Vaginal Delivery
WHO: World Health Organization
AOR: Adjusted Odds Ratio
COR: Crude Odds Ratio
CI: Confidence Interval
IQR: Interquartile Range
SD: Standard Deviation
