# Supplementary material for: The impact of the Tigray War on the incidence of low birth weight in Tigray, Ethiopia: A retrospective cohort study
Source: PLOS Glob Public Health. 2026 Aug 10;6(8):e0006298. doi: 10.1371/journal.pgph.0006298 (PMC13456257; doi:10.1371/journal.pgph.0006298)
Supplement: Supplementary file 2 [file pgph.0006298.s003.pdf]

Response to Reviewers

Editor's Comment

Journal Requirements:

1. Please provide additional details regarding participant consent. In the ethics statement in the Methods and online submission information, please ensure that you have specified (1) whether consent was informed and (2) what type you obtained (for instance, written or verbal, and if verbal, how it was documented and witnessed). If your study included minors, state whether you obtained consent from parents or guardians. If the need for consent was waived by the ethics committee, please include this information. If you are reporting a retrospective study of medical records or archived samples, please ensure that you have discussed whether all data were fully anonymized before you accessed them and/or whether the IRB or ethics committee waived the requirement for informed consent. If patients provided informed written consent to have data from their medical records used in research, please include this information.
   - **Response 1:** We have updated the Ethics Statement. The requirement for informed consent was waived by the Institutional Review Board (IRB) of Mekelle University (Ref: MU-IRB 1957/2022) as the study was a retrospective review of secondary medical records. All data were fully anonymized and de-identified prior to analysis; no personal identifiers (such as names or specific ID numbers) were accessed or recorded by the research team.

     Location of change: Methods (Ethics Statement), Page 5, Lines 87–95.
2. Please provide separate main figure files in .tif or .eps format only and ensure that all files are under our size limit of 10MB.
   - **Response 2**: Done. A high-resolution .tif file (300 DPI) has been prepared and uploaded separately.
3. Please include a separate legend or caption for Figure 1 in your manuscript.
   - **Response**: Done. The legend for Figure 1 has been included at the end of the manuscript file.

Reviewers' comments:

Reviewer's Responses to Questions

Reviewer #1: This is an interesting and important study. I compares lbw over four consequent year without and then with war in Tigray. I have have some questions and suggestions for a revision, mainly minor.

Comment 1: Background: In order to fully grasp the study and results I would have liked to understand the crude birth rate and the proportion of women who delivers in hospitals in the region (normally).

- **Response 1**: We thank the reviewer for this suggestion to provide context. Prior to the war, the Total Fertility Rate (TFR) in Tigray was 3.7. The proportion of institutional deliveries was approximately 81%. However, during the conflict, the regional health system faced severe disruption, with reports indicating that over 75% of health facilities were rendered non-functional. Because our study was conducted in Mekelle, the regional capital, we were able to access records from hospitals that remained partially functional as referral hubs throughout the crisis. This context has been added to the Introduction.
  Location of change: Introduction, Page 3, Lines 51–55.

Comment 2: Method: Sample the calculation of sample size is well explained. Out of how many in total? What was the proportion of excluded records?

- **Response 2**: We thank the reviewer for this request for clarification. Based on the sampling intervals used (k=16 for Ayder and k=20 for Mekelle General), the estimated total source population in the delivery registration books was approximately 31,734 deliveries over the four-year study period (~15,912 pre-war and ~15,822 during the war).
  Regarding exclusions, we initially calculated a required sample size of 1,526. To account for potential data quality issues common in retrospective chart reviews, particularly in conflict settings, we added a 15% allowance (237 records) for missing variables or lost

cards. Therefore, the analyzed sample of 1,763 reflects the final usable dataset after these exclusions were processed. We have added these details to the Methods section.

Location of change: Methods (Population and Sampling Technique), Page 5 & 6, Lines 103–116.

Comment 3: What was the systematic sampling technique used? How were the samples selected?

- **Response 3**: We have provided additional detail on the systematic sampling procedure. Within each hospital and for each of the two study periods, a sampling interval (k) was determined by dividing the total deliveries by the required sample size (k=16 for Ayder Comprehensive Referral Hospital and k=20 for Mekelle General Hospital). A random starting point (the first record) was selected using a lottery method, and then every k-th record was extracted until the required sample size for that period and facility was achieved.

  Location of change: Methods (Population and Sampling Technique), Page 6, Lines 111–116.

Comment 4: I would not call a data extraction tool a questionnaire - rather a data extraction form.

- **Response 4**: We agree with the reviewer. We have replaced the term data extraction tool a questionnaire with data extraction form throughout the manuscript to accurately reflect the retrospective nature of the data collection.

  Location of change: Methods (Data Extraction Form), Page 7, Lines 132.

Comment 5: Language: you write that. logistic regression is commonly used ...yes, but did you use it in your study?

- **Response 5**: We apologize for the ambiguity in the previous phrasing. We have clarified that multivariable binary logistic regression was the primary statistical method used in this study to calculate Adjusted Odds Ratios (AOR) and identify independent predictors of low birth weight while controlling for potential confounders.
- Location of change: Methods (Data Analysis), Page 8, Line 145-154.

Comment 6: Results: clear and well explained. Do you need to do normality testing logistic regression - I did not think that was relevant.

- **Response 6**: We agree with the reviewer that normality is not an assumption for logistic regression. The normality testing (Shapiro-Wilk) was originally performed only to describe the distribution of the continuous birth weight variable for descriptive statistics. To avoid confusion, we have removed the mention of normality testing from the sections describing the regression model requirements.
  Location of change: Methods (Data Analysis), Page 8, Line 145-154.

Comment 7: Discussion - well formulated and interesting comparisons and reasoning.

- **Response 7**: We thank the reviewer for the positive feedback regarding the depth and formulation of our Discussion.

Comment 8: In general, the language could be more "to the point" but overall fine.

- **Response 8**: We have revised of the entire manuscript for English/linguistic related issues. We have removed repetitive phrasing and tried to ensure the narrative is more concise and to the point as suggested.
